# Data on pre-service teachers’ experience of project activities based on the teacher education support project in Tanzania

**DOI:** 10.1016/j.dib.2022.108214

**Published:** 2022-04-25

**Authors:** Josephat Paul Nkaizirwa, Calvin Zakaria Swai, Alfred Kisuda Hugo, Cosmas Anyelwisye Mahenge, Philbert Sixbert Komba

**Affiliations:** a Department of Educational Psychology and Curriculum Studies, College of Education, the University of Dodoma, Tanzania; b The African Centre of Excellence for Innovative Teaching and Learning Mathematics and Science, College of Education, University of Rwanda, Rwanda; c Department of Mathematics and Statistics, College of Natural and Mathematical Sciences, the University of Dodoma, Tanzania; d The Teacher Training Section, Ministry of Education, Science and Technology, Tanzania

**Keywords:** Pre-service teachers, Professional learning, Teacher colleges, Teacher education, Teacher Education Support Project

## Abstract

Supporting teacher education in Tanzania has long been a common practice implemented by both local institutions and development partners. Despite a huge investment that has been dedicated to improve teacher education in Tanzania, a lot remains unclear on how direct beneficiaries perceive their engagement with the project activities including the milestones achieved by the implemented projects in teacher colleges (TCs). This article presents data on the experience of pre-service teachers (*N* = 2,772) participating in the Teacher Education Support Project (TESP), a project collaboratively implemented by the Governments of Tanzania and Canada. In this cross-sectional survey, data was collected from all the 35 public TCs in the Tanzania Mainland from May to August 2021. Exploratory factor analysis was conducted coupled with Monte-Carlo parallel analysis to examine the factor structure of the questionnaire alongside the descriptive analysis of pre-service teachers’ responses. The data covers four dimensions of the project services delivered to TCs, including library facilities, teaching and learning materials, science and ICT support as well as teaching and learning methods employed by tutors following TESP intervention. Broadly, useful insights that enlighten the progress made by the TESP so far are presented to stimulate the debate on how to successfully implement a development project geared towards strengthening teacher education in Tanzania and elsewhere. The presented data provides opportunity for educational researchers, teacher educators, policymakers, and curriculum developers to rethink on the key areas that need immediate attention to enhance the important work of preparing teachers in TCs in Tanzania and possibly beyond.

## Specifications Table

**Table:** 

Subject	Education
Specific subject area	Teacher education
Type of data	Table Figure
How the data were acquired	Data was collected from 2772 pre-service teachers using a semi-structured questionnaire from all of the public teacher colleges (TCs). Particularly, data was gathered through a self-administered questionnaire on the campuses of TCs in the Tanzania Mainland.
Data format	Raw Analyzed
Parameters for data collection	The aim of the present data was to explore the pre-service teachers’ experience of their engagement in the TESP implemented in all public TCs in the Tanzania Mainland. The data serves the purpose of describing the progress made by the project within a duration of three years since its inception. The data also highlights emerging suggestions to improve the project sustainability during and after its lifetime.
Description of data collection	Initially, the administered questionnaire was developed and modified to suit the purpose of data. Thereafter, research assistants were trained on the data collection protocol and processes followed by the actual fieldwork. Data collection took place from May 2021 to August 2021 followed by detailed data cleaning and analysis. The administered questionnaire contained closed and open-ended items.
Data source location	Data was collected from 35 TCs located in seven educational zones of the Tanzania mainland (see Fig. 1).
Data accessibility	The dataset titled “Survey of pre-service teachers at Government Teachers Colleges” is publicly available on: Repository name: Mendeley Data Repository Data identification number: DOI:10.17632/9xn2zgsxdj.1 Direct link to the dataset: https://data.mendeley.com/datasets/9xn2zgsxdj/1

## Value of the Data

• This data is useful to enlighten debate on the implementation of teacher education projects in TCs. In so doing, this data provides useful insights for rethinking effective strategies to ensure project sustainability in TCs not only in Tanzania but also in other similar contexts around the world.
• This data can help policymakers, teacher educators, researchers and curriculum developers on the key issues required to reshape the preparation of teachers in TCs. Specifically, the data highlights key areas that pre-service teachers have noted as critical, and therefore, require deliberate action and immediate attention to address.
• The data described herein helps to stimulate debates on how the project sustainability should be (re)considered and thought of even when the duration of the project comes to an end. The dataset is useful for shedding new light on subsequent researches that need to pick from where the presented dataset points an urgent need of attention to hearten teacher education in Tanzania.

## 1 Data Description

Implementation of teacher education projects has been widely documented in Tanzania. Some of such projects have been implemented to enhance professional development for either teachers working as in-service professionals or as pre-service teachers in TCs [1,2]. Observations from previously implemented teacher education projects indicate an increasing rate of unsustainability when a project duration ends. The TESP, however, is a unique project implemented in Tanzania from its design and implementation stages. On the former, the project covers the whole of the target population (all public TCs). On the Latter, the project involved a combination of actors in its implementation to ensure ownership and sustainability. This is not to mention the decision to streamline project activities within the teacher education system. As such, collecting data to document how the project has progressed and the ways pre-service teachers feel about its implementation was a useful consideration.

The data presented here was collected through semi-structured questionnaires on four dimensions, which are: library services and facilities, science and ICT materials, in-class teaching facilities as well as teaching and learning methods employed by college tutors as perceived by their pre-service teachers. The questionnaire was designed in accordance with the project targets in line with the teacher education curriculum of Tanzania [3]. The actual item wording is found in the shared file in Mendeley repository labeled as “Survey of pre-service teachers at Government Teachers available publicly through: https://data.mendeley.com/drafts/9xn2zgsxdj

**Fig. 1:**
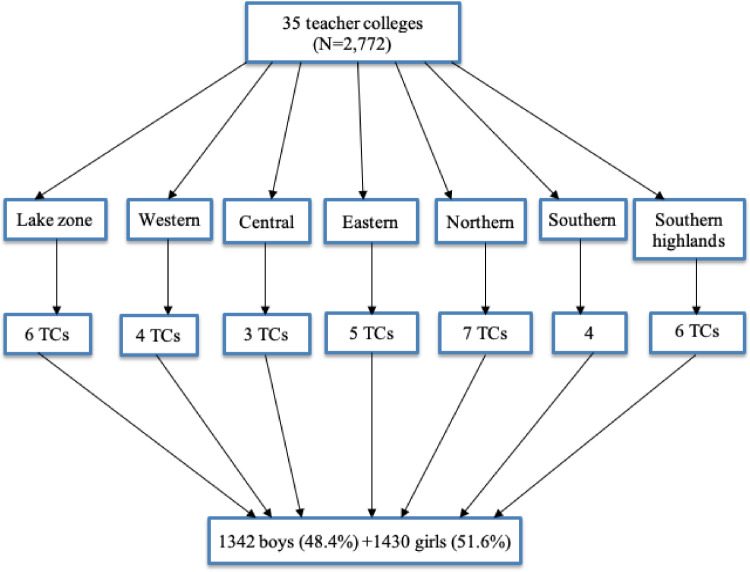
A graphical presentation of sample size and areas of data collection.

*Note*: TCs = Teacher colleges.

Analyses were conducted to explore the factor structure of the questionnaire followed up by descriptive and inferential statistics of data in accordance with pre-service teachers’ responses they provided to the questionnaire. Besides, demographic data associated with sex of respondents, academic year of study and the college location were collected simultaneously. The data was subjected to extensive scrutiny to reveal their suitability to the participants from whom they had been taken from. Reliability tests of the extracted factors were assessed using Cronbach's alpha of coefficient supported by corrected item-total correlation [4] as indicated in Table 1. The dataset file containing raw data is appended as a supplementary sheet (CSV) named Survey of pre-service teachers at Government Teachers available at “https://data.mendeley.com/drafts/9xn2zgsxdj” Fig. 1.

**Table 1:** Exploratory factor analysis for pre-service teachers (*N* = 2772).

Variables	Component				Cronbach (α)	CITC
	1	2	3	4		
IC 2	.817				.868	.754
IC 3	.787					.709
IC 1	.785					.731
IC 4	.770					.684
ICT 3		.834			.859	.745
ICT 2		.818				.728
ICT 1		.765				.676
ICT 4		.746				.668
TM 3			.790		.827	.669
TM 4			.770			.654
TM 2			.769			.656
TM 1			.745			.637
TEXT 1				.799	.808	.662
TEXT 2				.770		.621
TEXT 3				.737		.630
TEXT 4				.676		.590
Explained variance	17.66%	17.51%	16.71%	15.94%	Total explained variance: 67.82%	
Kaiser-Meyer-Olkin Measure of Sampling Adequacy (KMO)					.851	
Bartlett's Test of Sphericity						
*Approx. Chi-Square (r^2^)*					14,607.571	
*Df*					66	
*Sig.*					.000	

*Note*: CITC = Corrected Item-total correlation, df = Degree of freedom, IC = In-class resources, ICT = Information and communication technology, TM = Teaching methods.

## 2 Experimental Design, Materials and Methods

A cross-sectional survey design was used to collect data from pre-service teachers. The design allowed collection of data once at a point [5]. Before data collection, a designed questionnaire went through different stages of validation. Initially, the items were developed based on the performance measurement framework (PMF) of the project followed by the face validity of each item by three experts from the Ministry of Education, Science and Technology of Tanzania, the TESP Senior Advisor (one member), university academicians (five members), experts in monitoring, evaluation, and learning as well as educational researchers. Thereafter, the tool was piloted to equivalent participants before the actual data collection was conducted in the field. This validation reduced the number of items from 20 to 16 that met the required psychometric features. Further scrutiny of the tool for construct and criterion validity as well as internal consistency reliability was conducted for each of the items and for the factor structure of the scale.

Since the collection of data was conducted in all the public TCs, a simple random sampling was conducted to obtain a number of pre-service teachers from colleges. A total of 3000 respondents were obtained from seven zones. After collecting data, the screening process was conducted to filter out unrequired data from the data set created. Out of 3000 filled questionnaires, only 2772 (92.4%) were considered for subsequent analysis (Fig. 1).

The filled questionnaire items were subjected to exploratory factor analysis (EFA) to explore the factor structure of the scale based on the measured dimensions/constructs (library facilities and services, ICT and science facilities, effectiveness of teaching and learning methods employed by tutors as perceived by pre-service teachers, and in-class teaching and learning resources). The minimum item loading was set at 0.40 [6], eigenvalues greater than one were considered for determining the factor structure. Internal consistency reliability was computed accordingly for the whole scale and for each measured dimension. Monte-Carlo parallel analysis was conducted alongside the calculated eigenvalues for comparing the computed against the simulated eigenvalues for determining the number of factors to retain in the scale used for data collection.

For open-ended items, pre-service teachers were asked to provide any additional information regarding the areas they considered requiring improvement during the project implementation in TCs to enhance the project sustainability after the project lifetime. Inclusion of these items aimed at; first, revealing areas that needed immediate attention for the project to achieve its targets and second, to unfold the key factors to help other projects aiming at improving teacher education in Tanzania. Thematic description was conducted to group together related themes and presented in tables as multiple responses output data.

## Ethics Statements

The purpose for collecting data was communicated to the respondents in advance before collecting data. Ethics approval was not mandatory as the project evaluation was an ongoing activity under the Ministry of Education, Science and Technology. However, respondents’ consent and assent to fill the questionnaire was requested and granted by those who filled the questionnaire. Besides, respondents were informed that their responses were for the research purpose and they would not be used for personal gain and they were guaranteed confidentiality regarding their responses.

## CRediT authorship contribution statement

**Josephat Paul Nkaizirwa:** Conceptualization, Methodology, Data curation, Writing – original draft. **Calvin Zakaria Swai:** Conceptualization, Methodology, Writing – review & editing. **Alfred Kisuda Hugo:** Conceptualization, Methodology, Writing – review & editing. **Cosmas Anyelwisye Mahenge:** Conceptualization, Methodology, Data curation, Writing – review & editing. **Philbert Sixbert Komba:** Conceptualization, Methodology, Writing – review & editing.

## Declaration of Competing Interest

The authors declare that they have no known competing financial interests or personal relationships that could have appeared to influence the work reported in this paper.

## Data Availability

No data was used for the research described in the article.

## Appendix Supplementary Materials

Image, application 1

Image, application 2
