# Supplementary material for: Data on pre-service teachers’ experience of project activities based on the teacher education support project in Tanzania
Source: Data Brief. 2022 Apr 25;42:108214. doi: 10.1016/j.dib.2022.108214 (PMC9065589; doi:10.1016/j.dib.2022.108214)
Supplement: Supplementary file 1 [file mmc1.docx]

________________________________________________________________________________

**Survey of Student Teachers at Government Teacher Colleges**

| *This survey gathers information that will help the Teacher Education Support Project and the Teacher Education Unit of the Ministry of Education, Science and Technology to plan for the ongoing enhancement of Teacher Colleges and teacher education programs. Please fill the questionnaire items based on your own experience at your college over the past two years. Your cooperation is appreciated and your suggestions welcome.* |
| --- |

1. Name of the Teacher College you attend: ......................................................................................
2. Program of study:

Diploma Certificate

1. Subjects or area of specialization (For Diploma): ......................................................................................................................................

1. Gender: Male Female

**Each question in this survey asks you to give a rating by choosing a number between 1 and 5 on a five-point scale. *Please circle the number that best reflects your opinion.***

## **Textbooks, reference books and other resource material**

## Over the past two years, *the quality and quantity* of the textbooks, reference books, journals and other resource material available in the library and other places on campus to support my learning has:

| 5 | 4 | 3 | 2 | 1 |
| --- | --- | --- | --- | --- |
| Increased a lot | Increased | Stayed the same | Decreased | Decreased a lot |

- 1. Over the past two years, the use of textbooks, reference books, journals and other reference material has:

| 5 | 4 | 3 | 2 | 1 |
| --- | --- | --- | --- | --- |
| Increased a lot | Increased | Stayed the same | Decreased | Decreased a lot |

- 1. Since I began my studies at this college, my level of satisfaction with the quality and quantity of textbooks, reference books, journals and other reference material available to me has:

| 5 | 4 | 3 | 2 | 1 |
| --- | --- | --- | --- | --- |
| Increased a lot | Increased | Stayed the same | Decreased | Decreased a lot |

- 1. Overall, my level of satisfaction with the quality and quantity of textbooks, reference books, journals and other reference material available to me to support my learning is:

| 5 | 4 | 3 | 2 | 1 |
| --- | --- | --- | --- | --- |
| Very satisfied | Satisfied | Neutral | Dissatisfied | Not at all satisfied |

- 1. My suggestion for textbooks, reference books and resource materials to improve the quality of learning are: ....................................................................................................................................................................................................................................................................................................

## **Computers and other information and communication technology (ICT) hardware and software**

## Over the past two years, the quality and quantity of computers, computer programs and on-line learning resources available in ICT labs, the library and classrooms to support my learning has:

| 5 | 4 | 3 | 2 | 1 |
| --- | --- | --- | --- | --- |
| Increased a lot | Increased | Stayed the same | Decreased | Decreased a lot |

- 1. Over the past two years, *my use* of computers, computer programs and on-line learning resources has:

| 5 | 4 | 3 | 2 | 1 |
| --- | --- | --- | --- | --- |
| Increased a lot | Increased | Stayed the same | Decreased | Decreased a lot |

- 1. Since I began my studies at this college, my level of satisfaction with the quality and quantity of computers, computer programs and on-line learning resources available to me has:

| 5 | 4 | 3 | 2 | 1 |
| --- | --- | --- | --- | --- |
| Increased a lot | Increased | Stayed the same | Decreased | Decreased a lot |

- 1. Overall, my level of satisfaction with the quality and quantity of computers, computer programs and on-line learning resources to me to support my learning is:

| 5 | 4 | 3 | 2 | 1 |
| --- | --- | --- | --- | --- |
| Very satisfied | Satisfied | Neutral | Dissatisfied | Not at all satisfied |

- 1. My suggestion for computers, computer programs and on-line learning resources to improve the quality of learning are: ....................................................................................................................................................................................................................................................................................................

## **In-class teaching and learning aids and practical materials (such as maps, charts, white boards, science kits, mathematics sets, laboratory supplies, models, specimens, etc.)**

- 1. Over the past two years, the quality and quantity of teaching and learning aids and practical materials available in classrooms and laboratories to support my learning has:

| 5 | 4 | 3 | 2 | 1 |
| --- | --- | --- | --- | --- |
| Increased a lot | Increased | Stayed the same | Decreased | Decreased a lot |

- 1. Over the past two years, my tutors’ use of teaching and learning aids, practical materials and laboratory supplies to enhance teaching and learning has:

| 5 | 4 | 3 | 2 | 1 |
| --- | --- | --- | --- | --- |
| Increased a lot | Increased | Stayed the same | Decreased | Decreased a lot |

- 1. Since I began my studies at this college, my level of satisfaction with the quality and quantity of teaching and learning aids, practical materials and laboratory supplies available and used in-class has:

| 5 | 4 | 3 | 2 | 1 |
| --- | --- | --- | --- | --- |
| Increased a lot | Increased | Stayed the same | Decreased | Decreased a lot |

- 1. Overall, my level of satisfaction with the quality and quantity of teaching and learning aids, practical materials and laboratory supplies is:

| 5 | 4 | 3 | 2 | 1 |
| --- | --- | --- | --- | --- |
| Very satisfied | Satisfied | Neutral | Dissatisfied | Not at all satisfied |

- 1. My suggestion for teaching and learning aids, practical materials and laboratory supplies to improve the quality of learning are: ....................................................................................................................................................................................................................................................................................................

## **Teaching Methods (lectures, presentations, discussions, demonstrations, group work, experiments, field studies, practical exercises, projects, assessment, etc.)**

- 1. Over the past two years, the teaching methods used by my Tutors to help me learn have become:

| 5 | 4 | 3 | 2 | 1 |
| --- | --- | --- | --- | --- |
| More effective | Effective | Stayed the same | Less effective | Not effective |

- 1. Over the past two years, the methods my tutors use to ensure that all students in my classes are equitably engaged in learning (males and females, students with disabilities, students from different communities), have become:

| 5 | 4 | 3 | 2 | 1 |
| --- | --- | --- | --- | --- |
| More effective | Effective | Stayed the same | Less effective | Not effective |

- 1. Since I began my studies at this college, my level of satisfaction with the quality and equity of my Tutors’ teaching methods has:

| 5 | 4 | 3 | 2 | 1 |
| --- | --- | --- | --- | --- |
| Increased a lot | Increased | Stayed the same | Decreased | Decreased a lot |

- 1. Overall, my level of satisfaction with the quality and equity of my Tutors’ teaching methods is:

| 5 | 4 | 3 | 2 | 1 |
| --- | --- | --- | --- | --- |
| Very satisfied | Satisfied | Neutral | Dissatisfied | Not at all satisfied |

- 1. My suggestion for teaching methods to improve the quality of learning are: ....................................................................................................................................................................................................................................................................................................
